# A Facile Ginger Straw-Based Self-Nitrogen-Doped Biochar Activated by NaHCO_3_: Fast and Efficient Adsorption Toward Food Dyes of Tartrazine and Carmine

**DOI:** 10.3390/foods15152668

**Published:** 2026-07-29

**Authors:** Mingwan Liu, Zhuhua Gong, Zhenghang Guo, Yu Yu, Shuangshuang Bai, Qi Zhang, Qinhong Liao, Hongjia Lu, Honglei Li, Yuming You, Wenlin Zhang

**Affiliations:** Chongqing Key Laboratory for Germplasm Innovation of Special Aromatic Spice Plants, College of Smart Agriculture/Institute of Special Plants, Chongqing University of Arts and Sciences, Chongqing 402160, China; 15736066449@163.com (M.L.); 15208482161@163.com (Z.G.); g7z1h2000@163.com (Z.G.); 15696432690@163.com (Y.Y.); 17364958959@163.com (S.B.); 18183376283@163.com (Q.Z.); lqhwisdom@126.com (Q.L.); aaluhongjia@163.com (H.L.); lihonglei215@163.com (H.L.)

**Keywords:** carmine, tartrazine, ginger straw, biochar, density functional theory

## Abstract

Food industry wastewater containing synthetic dyes threatens ecological safety and human health. Therefore, efficient and environmentally friendly adsorbents that can remove synthetic food dyes from wastewater are urgently needed. In this work, a self-nitrogen-doped biochar (GSNBC), used for adsorption of food dyes including carmine and tartrazine, was facilely prepared by employing ginger straw waste as the carbon precursor and NaHCO_3_ as a mild and relatively benign pore-forming agent via one-step pyrolysis. The as-prepared GSNBC featured well-developed porous structures, a specific surface area of 1712.52 m^2^ g^−1^, and rich oxygen- and nitrogen-containing functional surface groups. Particularly, GSNBC performed ultrafast adsorption, with approximately 90% of the equilibrium capacity within 1 min (600.13 mg g^−1^ and 580.24 mg g^−1^ for carmine and tartrazine, respectively), and reached adsorption equilibrium at about 10 min. In addition, it exhibited excellent regenerability. The adsorption kinetics and isotherms fit well with pseudo-second-order and Langmuir models. DFT calculations indicated that π–π stacking and hydrogen bonding were mainly responsible for the adsorption. This study presents not only a promising and efficient adsorbent for the remediation of dye-laden food industry wastewater but also a sustainable route for the resource utilization of ginger straw waste.

## 1. Introduction

Food dyes are additives used to improve the visual appeal of food products by enhancing their attractiveness and stimulating appetite. Currently, approximately 11 synthetic food dyes such as tartrazine, carmine, sunset yellow, amaranth, ponceau, allura red, and brilliant blue, etc., are used worldwide. Among them, tartrazine and carmine are the most commonly used in the food industry [1]. Tartrazine is an anionic azo dye widely used in baked goods, desserts, candies, condiments, spreads, jellies, soft drinks, and canned foods to impart a bright yellow hue [2]. Carmine, a natural dye extracted from female cochineal insects, is used in yogurt, beverages, candies, and meat products for its deep red coloration [3]. When discharged into surface or groundwater, these dyes not only endanger aquatic and terrestrial ecosystems by causing severe water pollution but may also undergo biotransformation into aromatic amine-mutagenic precursors that induce health risks such as hyperactivity, asthma, and thyroid cancer [1]. Therefore, the development of efficient, cost-effective, and environmentally benign technologies to remove such dyes from food and beverage industry effluents is crucial for safeguarding ecological systems and human health.

A wide range of strategies have been proposed to treat dye-containing wastewater., including precipitation, coagulation, filtration, reverse osmosis, oxidation, adsorption, and ion exchange [4]. Nevertheless, most of these methods have generally high costs, complicated processing, and unsatisfactory removal performance. By contrast, adsorption is widely used owing to its operational simplicity, low cost, and high removal efficiency. Various adsorbent materials have been reported, including clay minerals, polymers, metal–organic frameworks, metal nanoparticles, and their composites [5]. However, they have limited specific surface areas and poor regeneration performance and are expensive [6]. Biochar has garnered considerable attention for the treatment of dye wastewater owing to its straightforward preparation, abundant feedstock availability, and low cost. Studies have shown that biochar exhibits favorable adsorption performance toward synthetic dyes, including indigo [7], tartrazine [7], crystal violet [8], and sunset yellow [9]. In recent years, the preparation of biochar derived from agricultural and forestry residues, such as straw, rice husks, fruit shells, kernels, and bamboo [10], for wastewater treatment has become a research focus. Among various preparation strategies, chemical activation is the predominant method, where KOH, ZnCl_2_, K_2_CO_3_, and H_3_PO_4_ are commonly used activators [11]. Although the resulting biochars typically possess abundant pores and high specific surface areas, they suffer from drawbacks such as severe equipment corrosion and environmental pollution. By contrast, NaHCO_3_ is a milder and greener alternative. For example, Zhang [12] prepared biochar from cassava ethanol sludge via NaHCO_3_ activation, achieving a tetracycline adsorption capacity of 154.46 mg g^−1^. Zhao [13] used NaHCO_3_ to activate loofah-derived carbon materials, greatly enhancing the tetracycline adsorption capacity to 520.91 mg g^−1^. Wu [6] synthesized a porous manganese–nitrogen codoped biochar via NaHCO_3_ activation, which displayed a bisphenol A adsorption capacity of 351.13 mg g^−1^. Hence, developing porous biochar derived from agricultural and forestry residues using NaHCO_3_ as a milder activator for efficient dye adsorption represents a promising direction for treating food dye-contaminated wastewater.

However, pristine biochar is inherently hindered by its underdeveloped pore structure, limited surface-active sites, and insufficient selective adsorption capacity, which severely restrict its application in complex organic wastewater treatment. Nitrogen functionalization is widely recognized as a highly effective strategy for improving the adsorption performance of biochar. The incorporation of N atoms not only modulates the electronic structure and surface chemical properties of the carbon matrix but also generates abundant active sites, such as pyridinic N (N-6), pyrrolic N (N-5), and graphitic N. This structural evolution consequently strengthens multiple adsorption mechanisms, including electrostatic interactions, hydrogen bonds, π–π interactions, and Lewis acid–base interactions [14]. In particular, for azo-type food dyes characterized by aromatic conjugated structures and ionizable groups, an N-enriched surface significantly improves both adsorption capacity and binding affinity. Compared with exogenous nitrogen doping, in situ self-nitrogen doping presents distinct advantages, including simplified processing, higher feedstock utilization efficiency, and reduced environmental risks, as it enables the direct construction of N-containing functional groups during the pyrolysis process. Therefore, selecting naturally N-abundant biomass precursors coupled with mild activation strategies to fabricate porous N-rich biochar is a highly feasible and promising approach for the highly efficient removal of food dyes.

**Figure 1:**
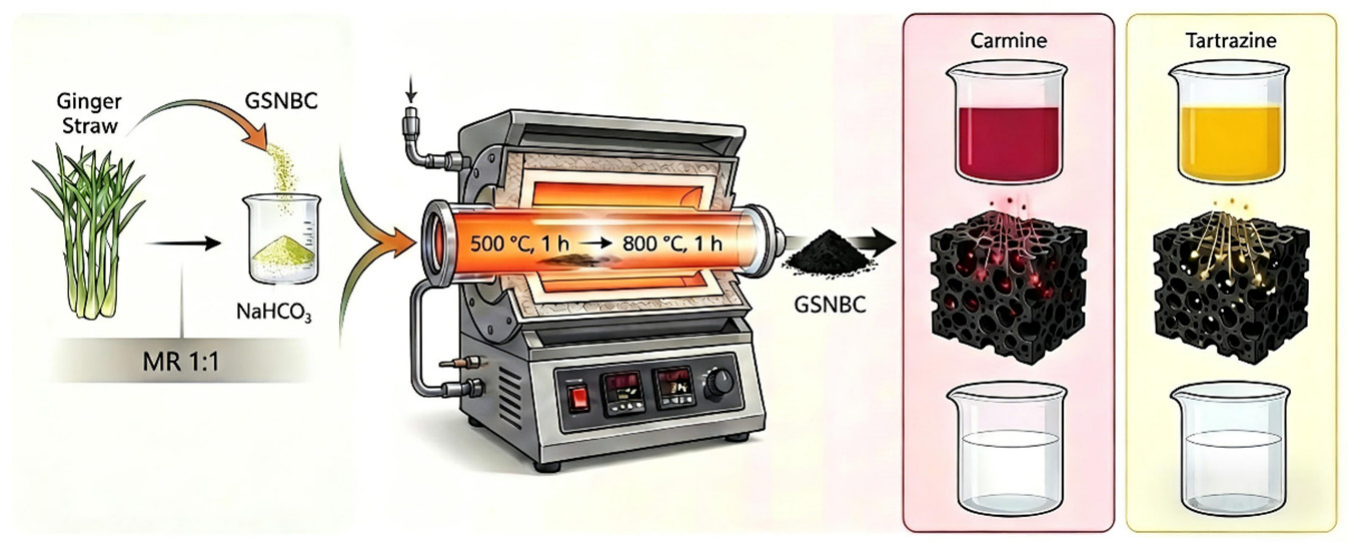
Schematic diagram of GSNBC preparation and adsorption toward carmine and tartrazine.

Ginger (*Zingiber officinale* Roscoe) is a globally acclaimed dual-purpose plant with medicinal and edible value. Its subterranean rhizomes are extensively valorized across the food, pharmaceutical, and beverage industries [15]. Paradoxically, the aerial part, ginger straw, is routinely discarded as agricultural refuse, triggering severe environmental pollution and tremendous resource squandering. While previous valorization attempts have converted ginger straw into briquettes, biogas, and bioethanol, these conventional approaches are substantially hindered by low resource conversion efficiencies and marginal economic viability [15]. Ginger straw, characterized by its inherently robust and dense lignocellulosic matrix, serves as a promising biomass precursor for biochar production. Although Zhang [16] synthesized ginger straw-derived biochar via direct oxygen-limited pyrolysis, its specific surface area and dye adsorption affinity remained suboptimal, leaving ample room for structural enhancement. Consequently, employing a mild and cost-effective activating agent such as NaHCO_3_ to engineer ginger straw biochar with high specific surface area and dye adsorption efficiency has emerged as a highly compelling strategy. Motivated by these insights, this study pioneered the fabrication of a ginger straw-based self-N-doped biochar (GSNBC) via a facile NaHCO_3_-assisted activation route for fast and efficient adsorption of food dyes including carmine and tartrazine (Figure 1). Furthermore, density functional theory (DFT) calculations were integrated to decode the underlying adsorption mechanisms at the molecular level, providing profound theoretical guidance for the targeted removal of complex food dyes.

## 2. Materials and Methods

### 2.1. Materials and Chemicals

Ginger straw was collected from an experimental ginger field in Yongchuan, Chongqing, China. Carmine and tartrazine sodium bicarbonate (NaHCO_3_) was provided by Shanghai Aladdin Biochemical Technology, based in Shanghai, China; anhydrous ethanol (C_2_H_5_OH), HCl, and NaOH, all of analytical grade, were supplied by Chengdu Kelong Chemical Reagent (Chengdu, China). Purified water was used in all experiments.

### 2.2. Preparation of GSNBC

The raw ginger stalk material was cleaned to remove impurities and was sun-dried, pulverized, and sieved through a 60-mesh screen. Then, 2 g of ginger straw/NaHCO_3_ mass ratios of 1:0, 1:0.5, 1:1, and 1:2 and the mixtures were placed in a tube furnace. Under N_2_ protection, the samples were heated to 300 °C at 2 °C min^−1^ and held for 1 h, then further heated to 800 °C at 5 °C min^−1^ and held for another 1 h. After natural cooling, the crude biochar was ground, washed twice with 1 mol L^−1^ HCl under ultrasound, and then once with purified water under ultrasound to remove impurities. Subsequently, the samples were dried to constant weight in an air-blast drying oven at 50 °C.

### 2.3. Characterization of GSNBC

The physicochemical properties of the materials were analyzed using a field-emission scanning electron microscope (SEM, Hitachi SU-4800, Tokyo, Japan), a transmission electron microscope (TEM, FEI Tecnai G2 F20, Hillsboro, OR, USA) fitted with an energy-dispersive X-ray spectrometer (EDS), a surface area and porosity analyzer (Micromeritics ASAP 2460, Norcross, GA, USA), an X-ray diffractometer (XRD, Bruker D8 Advance, Karlsruhe, Germany), a confocal microscope–laser Raman spectrometer (Horiba Scientific LabRAM HR Evolution, Edison, NJ, USA), a Fourier-transform infrared (FTIR) spectrometer (Nicolet 6700, Madison, WI, USA), and an X-ray photoelectron spectrometer (XPS, Thermo Escalab 250 Xi, Waltham, MA, USA).

### 2.4. Adsorption Experiment of GSNBC Toward Dyes

#### Batch Adsorption

The experiment began by adding 10 mg of GSNBC to 10 mL of the dye solution, followed by shaking at 200 rpm at varying pH values (2–10), initial dye concentrations (*c*_0_: 100–700 mg L^−1^), adsorption times (t: 0.5 to 160 min), and temperatures (T: 25–45 °C). After adsorption, the mixture was centrifuged at 5000 rpm for 10 min and the supernatant was collected. A UV–Vis spectrophotometer (Beijing Persee General Instrument, Beijing, China) was used to measure the absorbance of the supernatant at 508 and 428 nm. The residual dye concentrations were calculated using the standard curves of carmine and tartrazine. Each sample was measured in triplicate to ensure accuracy and stability of the experiments [17]. Statistical analyses were conducted using IBM SPSS Statistics 26.0. All data are expressed as mean ± SD of triplicate measurements. Two-way ANOVA was used to assess the effects of temperature, initial concentration, adsorption time, and their interactions on adsorption capacity. Post -hoc comparisons were performed using Tukey’s HSD test when the main effect was significant (*p* < 0.05), with α = 0.05. The adsorption capacities and removal rates were calculated using the following equation:

(1) $$qe=\frac{(c_{0}-c_{e})\times v}{m}$$

where *q*_e_ (mg g^−1^) is the equilibrium adsorption capacity of GSNBC for the dye; *c*_0_ (mg L^−1^) and *c*_e_ (mg L^−1^) are the initial and equilibrium dye concentrations, respectively; *v*(L) is the solution volume; and *m*(g) is the adsorbent mass.

### 2.5. Recyclability and Potential Application of GSNBC

Regeneration experiments were conducted by adding 10 mg GSNBC to 10 mL of dye (700 mg L^−1^, pH 2), shaking at 200 rpm, 25 °C for 60 min, centrifuging at 5000 rpm for 10 min, and measuring the supernatant absorbance. Anhydrous ethanol was then added to the recovered GSNBC, and the mixture was shaken for 10 min for desorption, followed by centrifugation to remove the supernatant. The desorbed GSNBC was used in subsequent adsorption cycles. This adsorption–desorption cycle was repeated five times. For the practical application, the real food industry wastewater was spiked with 100 mg L^−1^ of each dye, and the adsorption experiment was conducted following the above-mentioned adsorption procedure.

### 2.6. DFT Calculations

All quantum-chemical calculations were performed with Gaussian 16 (C.01). Ground-state geometries of all molecules and complexes were fully optimized at the B3LYP-D3(BJ) level, i.e., B3LYP augmented by Grimme’s D3 dispersion with Becke–Johnson damping. The 6-31g(d) basis set was used for all atoms. The Solvation Model was included in both the geometric optimizations (water as dielectric). Wavefunction analyses (IGMH) were performed with Multiwfn 3.8 (dev), and molecular graphics were prepared using VMD.

## 3. Results

### 3.1. Preparation and Characterization of GSNBC

**Figure 2:**
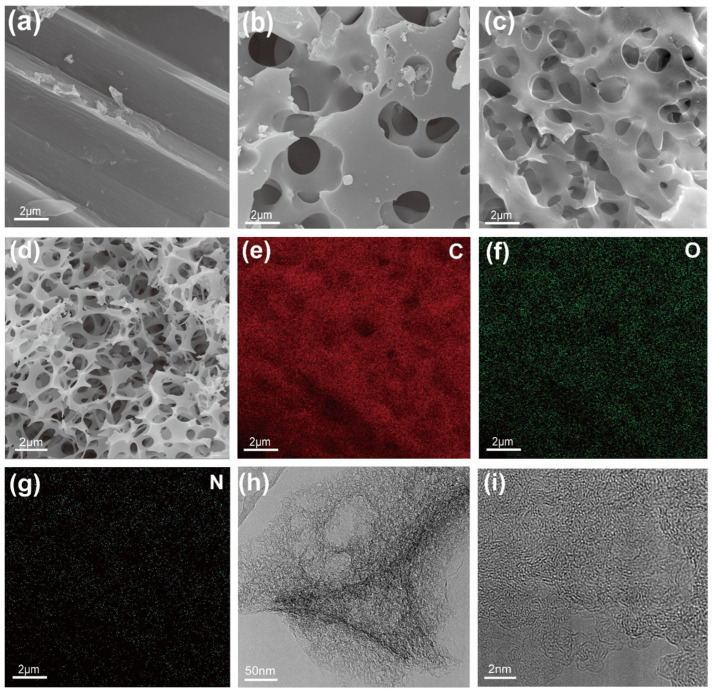
Scanning electron microscope (SEM) images of GSNBC-0.5 prepared at ginger straw/NaHCO_3_ mass ratios of 1:0 (**a**), 1:0.5 (**b**), 1:1 (**c**), and 1:2 (**d**), energy-dispersive X-ray spectrometer (EDS) mapping (**e**–**g**) and transmission electron microscope (TEM) (**h**,**i**) of GSNBC.

In this study, GSNBC was prepared via a one-pot method using ginger stalks as the raw material and NaHCO_3_ as the activator. The biochar obtained without NaHCO_3_ exhibited a relatively smooth surface with few pores and a compact, dense morphology (Figure 2a). After introducing NaHCO_3_, the resulting GSNBC samples displayed obvious microporous structures (Figure 2b–d). When the NaHCO_3_ addition ratio reached 1:1, GSNBC exhibited a rich and uniformly distributed microporous network (Figure 2c) with clear and well-interconnected channels, which is highly conducive to the migration of dye molecules toward the material surface and interior, further enhancing its adsorption performance [18]. However, when the NaHCO_3_ addition ratio was further increased to 2:1 (Figure 2d), the pores became excessively enlarged, accompanied by local structural collapse, indicating that over-etching of the carbon skeleton by the excessive activator compromised the mechanical stability of the material. According to the pore structure, analyzed by scanning electron microscopy, as well as the adsorption performance (Figure S1), GSNBC prepared at a NaHCO_3_ addition ratio of 1:1 exhibited an optimal structure and performance. Moreover, the EDS results confirmed that GSNBC contained abundant C, O, and N uniformly distributed within the 3D carbon framework (Figure 2e–g), which is beneficial for enhancing dye adsorption [19]. TEM images of GSNBC revealed abundant micropores and mesopores and an amorphous carbon morphology (Figure 2h,i).

**Figure 3:**
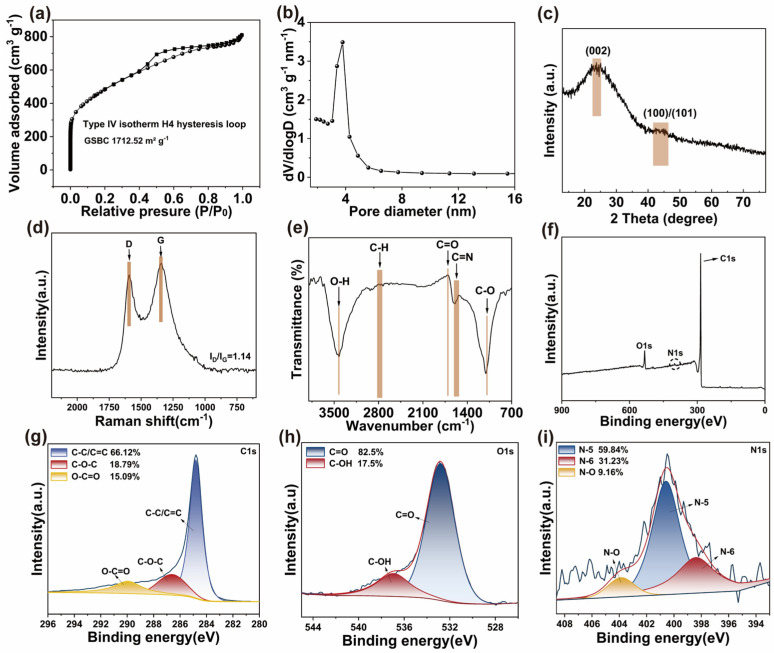
N_2_ adsorption–desorption isotherm (**a**), pore size distribution (**b**), X-ray diffractometer (XRD) pattern (**c**), Raman spectrum (**d**), Fourier-transform infrared (FTIR) spectrum (**e**) and X-ray photoelectron spectrometer (XPS) spectrum (**f**), high-resolution C 1s (**g**), O 1s (**h**), and N 1s (**i**) spectra of GSNBC.

The N_2_ adsorption–desorption isotherm of GSNBC (Figure 3a) was identified as a typical type IV isotherm according to the IUPAC classification [20]. At low relative pressures (P/P_0_ < 0.4), the N_2_ uptake increased sharply, indicating the presence of abundant micropores in GSNBC. In the range of 0.4 < P/P_0_ < 0.8, a hysteresis loop appeared, indicating that GSNBC was rich in mesopores. The pore size distribution curve (Figure 3b) revealed that GSNBC possessed both mesoporous and microporous structures, with pore sizes mainly centered between 1 and 5 nm. Abundant mesopores and micropores can effectively capture dye molecules, enhancing the dye-adsorption performance of the biochar toward dyes [21]. The specific surface area (*S*_BET_) and total pore volume (*V*_p_) of GSNBC were 1712.52 and 1.25 cm^3^ g^−1^, respectively, which were significantly higher than other reported agricultural and forestry residues, such as date palm petiole, sweet potato residue, sawdust and apricot stones-based carbon materials (Table S5). Moreover, its *S*_BET_ and *V*_p_ were 29.54- and 10.42-fold higher than those of the unactivated biochar (57.98 and 0.12 cm^3^ g^−1^, respectively; Table S1), demonstrating the high efficiency of NaHCO_3_ in pore creation and providing more adsorption sites for dye molecules. During the pore formation process, NaHCO_3_ thermally decomposed into Na_2_CO_3_ and CO_2_ at 100–200 °C (see Supporting Information S2.1), leading to the preliminary activation of the biochar. At 700–800 °C, Na_2_CO_3_ and its decomposition product, Na_2_O, further reacted with carbon, etching the carbon skeleton and generating pores [21]. Meanwhile, the sodium vapor, CO_2_, and CO gases produced during the reactions also contributed to pore structure formation and were subsequently carried away with the protective gas [22,23].

XRD analysis (Figure 3c) revealed that GSNBC is largely amorphous with a low graphitization degree, as evidenced by two broad diffraction peaks at 26° and 44° corresponding to the (002) and (100/101) planes of turbostratic carbon [24]. The Raman spectrum of GSNBC (Figure 3d) displays two characteristic bands, the D band at 1342 cm^−1^ and the G band at 1595 cm^−1^, reflecting the degree of disorder, defects, and graphitization. The density of defects and disorder in the biochar were positively correlated with the I_D_/I_G_ value, which was inversely proportional to the degree of graphitization [25]. The calculated I_D_/I_G_ value of 1.14 indicates a high degree of disorder and abundant defects, as well as a low graphitization level, which is consistent with the XRD and TEM results. These amorphous and defective structures are favorable for the adsorption of organic dyes [26].

The surface functional groups and elemental composition of GSNBC were analyzed using FTIR and XPS. As shown in Figure 3e, the broad and intense peak at 3427 cm^−1^ is attributed to the O–H stretching vibration, while the peak in the range of 1382–1385 cm^−1^ is associated with the C=N stretching vibration. The peaks at 1740 and 1039 cm^−1^ correspond to C=O and C–O stretching vibrations, respectively. The bands at 2924 and 2853 cm^−1^ correspond to the asymmetric and symmetric stretching vibrations of aliphatic C–H bonds, respectively [27], indicating that certain aliphatic chain structures were partially retained in the organic carbon skeleton after pyrolysis. These results demonstrate that the surface of GSNBC is rich in functional groups such as O–H, C=O, C=N, and C–O, which can promote the adsorption process through electrostatic attraction, hydrogen bonding, and coordination complexation with dye molecules [28]. The XPS survey spectrum displayed three distinct peaks at 285.0, 532.1, and 400 eV, assigned to C 1s, O 1s, and N 1s, respectively (Figure 3f), confirming the presence of C, O, and N in GSNBC, with contents of 91.79, 6.64, and 1.57%, respectively. Nitrogen originates from the transformation of nitrogen-containing organic compounds in the ginger stalks during pyrolysis [29]. In the high-resolution C 1s spectrum (Figure 3g), the peaks at 284.8, 286.9, and 288.5 eV are attributed to C–C/C=C (66.12%), C–O–C (18.79%), and O–C=O (15.09%), respectively [30]. The dominant peak at 284.8 eV, primarily associated with sp^2^ hybridization, indicates the existence of ordered π–π conjugated structures in GSNBC. The C–O–C and O–C=O sub-peaks correspond to the oxygen-bound amorphous carbon phases, which provide active hydrophilic coordination sites [31]. The O 1s spectrum (Figure 3h) displays two characteristic peaks at 532.1 and 536.5 eV, which are assigned to C=O (82.5%) and C–OH (17.5%), respectively. A high proportion of C=O groups is critical for enhancing the adsorption capacity of biochar, as it provides additional active centers for dye adsorption and strengthens the affinity of the material toward dye molecules [32]. In the N 1s spectrum (Figure 3i), the peaks at 400.1, 398.1, and 402.2 eV correspond to N-5 (59.84%), N-6 (31.23%), and N-O (9.16%) [33], respectively, indicating that nitrogen in GSNBC predominantly exists in the form of N-5. The N-5 species can enhance the hydrophilicity of the carbon framework through the -N-H groups in its five-membered heterocyclic structure and serve as electron donors to increase the local electron density, strengthening the adsorption capacity toward reactants [33]. The lone pair of electrons in N-6 can participate in the construction of Lewis basic sites, further enriching the diversity of the chemically active surface sites.

### 3.2. Adsorption Performance of GSNBC Toward Dyes

#### 3.2.1. Factors Influencing Adsorption

**Figure 4:**
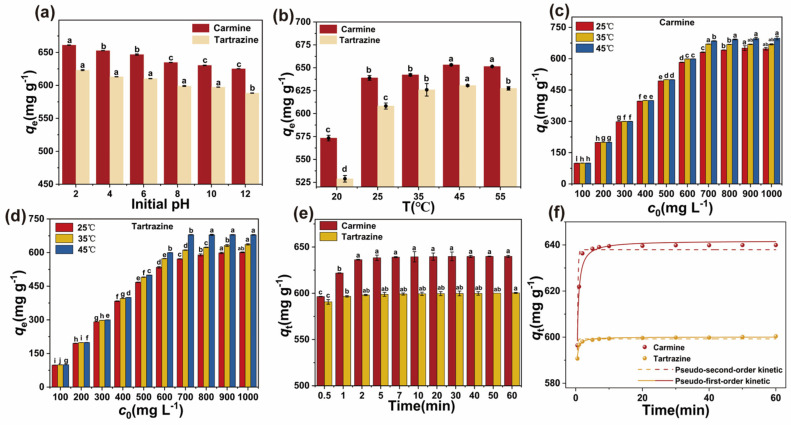
Effects of pH (*c*_0_: 700 mg L^−1^, T: 25 °C, t: 60 min) (**a**), *T* (*c*_0_: 700 mg L^−1^, pH: 2, t: 60 min) (**b**), *c*_0_ (pH: 2, T: 25 °C, t: 60 min) (**c**,**d**), *t* (*c*_0_: 700 mg L^−1^, pH: 2, T: 25 °C) (**e**,**f**) of carmine and tartrazine adsorption by GSNBC. Different letters within the same group indicate significant differences (*p* < 0.05).

Solution pH plays a crucial role in the adsorption behavior of biochar. As shown in Figure 4a, the *q*_e_ values of GSNBC toward carmine and tartrazine fluctuated little in a wide pH range. Moreover, at pH 12, GSNBC still exhibited high *q*_e_ values of 625.18 mg g^−1^ for carmine and 588.73 mg g^−1^ for tartrazine, which were only 36.04 mg g^−1^ and 34.84 mg g^−1^ lower than those at pH 2, respectively, indicating that electrostatic interaction was not the dominant mechanism and that other strong adsorption forces were involved. As shown in Figure 4b, the *q*_e_ values of GSNBC for carmine and tartrazine increased with increasing temperature, reaching 655.21 and 626.16 mg g^−1^ at 45 °C, respectively. This suggests that higher temperatures enhance dye diffusion in both the solution and the adsorbent, and may also strengthen chemical bonding with surface functional groups, thereby promoting adsorption [34]. With increasing initial dye concentration, the adsorption capacities of GSNBC for carmine and tartrazine also increased continuously (Figure 4c,d), demonstrating that the concentration gradient enhanced the mass-transfer driving force and drove more dye molecules to migrate toward the GSNBC surface [35]. When *c*_0_ ≥ 400 mg L^−1^, the adsorption capacity showed a slow saturation trend, as the effective adsorption sites on the GSNBC surface became nearly saturated. The equilibrium adsorption capacity increased with initial concentration at all temperatures, consistent with previous reports on carmine and tartrazine adsorption by sweet potato residue biochar [36]. The adsorption kinetics of carmine and tartrazine on GSNBC (Figure 4e) can be characterized by an initial rapid uptake (<1 min), followed by a gradual stage (1–10 min) and reaching equilibrium beyond 10 min. After 1 min, the adsorption capacities reached 600.13 and 580.24 mg g^−1^ for carmine and tartrazine, respectively. This ultrafast adsorption behavior can be ascribed to the strong mass-transfer driving force provided by the high concentration gradient at the initial stage, large number of unoccupied surface active sites, and low mass-transfer resistance arising from the hierarchical porous structure [37]. As the adsorption sites were gradually occupied, the adsorption rate decreased, and equilibrium was eventually reached at 10 min.

#### 3.2.2. Adsorption Kinetics

The adsorption kinetics of carmine and tartrazine onto GSNBC were evaluated via pseudo-first-order and pseudo-second-order models (Supporting Information S2.2, Figure 4f, and Table S2). The pseudo-second-order model gave *R*_2_ values of 0.973 (carmine) and 0.978 (tartrazine), clearly outperforming the pseudo-first-order model (*R*_2_ = 0.881; *R*_2_ = 0.881 and 0.852, respectively). Moreover, the adsorption capacities calculated from the pseudo-second-order model (*q*_e,cal_) agreed well with the experimental values (*q*_e,exp_), indicating that the pseudo-second-order model can more accurately describe the adsorption of both dyes onto GSNBC, revealing that chemisorptions existed between food dyes and GSNBC [38]. Furthermore, the adsorption rate constants (*k*_2_) of carmine and tartrazine were 0.44 and 0.22 g mg^−1^ min^−1^, respectively. This difference can be attributed to the distinct adsorption mechanisms of the two dyes: the higher rate constant of carmine is likely associated with its molecular structure, which contains more aromatic rings, facilitating the rapid establishment of π–π interactions with the sp^2^-C domains on the GSNBC surface. By contrast, tartrazine contains more sulfonic acid and azo groups, which result in greater steric hindrance and electrostatic repulsion during surface mass transfer, leading to a slower adsorption rate and increased mass-transfer resistance [39].

#### 3.2.3. Adsorption Isotherms

**Figure 5:**
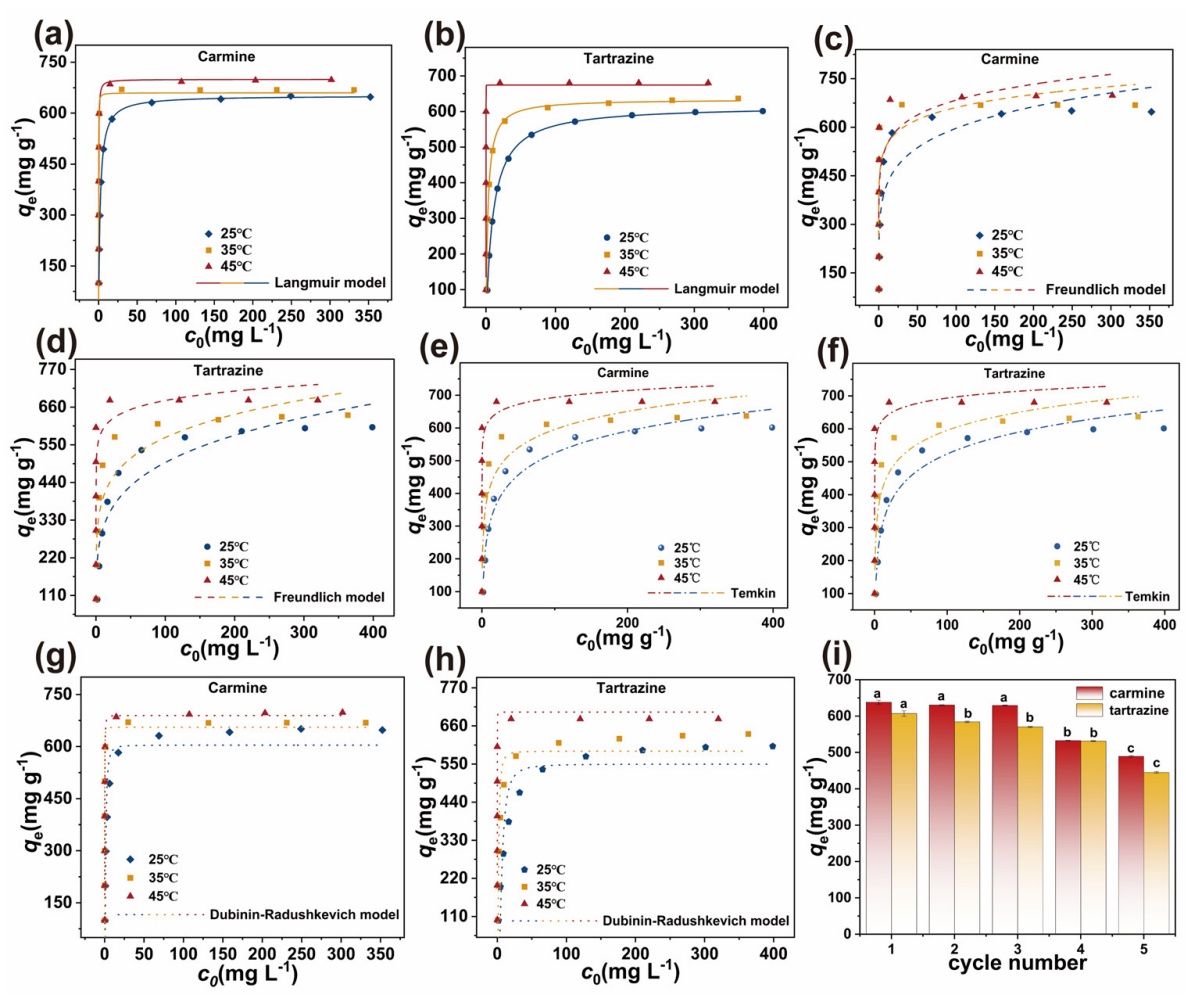
The Langmuir (**a**,**b**), Freundlich (**c**,**d**), Temkin (**e**,**f**), Dubinin–-Radushkevich (**g**,**h**) models, and cyclic regeneration (**i**) of carmine and tartrazine adsorption by GSNBC. Different letters within the same group indicate significant differences (*p* < 0.05).

Adsorption isotherm models are crucial for evaluating the performance of adsorbents and elucidating the underlying mechanisms (see Supporting Information S2.3). The adsorption process was analyzed and fitted using the Langmuir, Freundlich, Temkin, and Dubinin–Radushkevich models, and the corresponding results are presented in Figure 5a–h and Table S3. The Langmuir model exhibited high correlation coefficients (*R*^2^ = 0.941–0.999), which were markedly superior to those of the Freundlich model (*R*^2^ = 0.651–0.854), indicating that the adsorption process conforms to the Langmuir model and suggesting that monolayer adsorption predominates under the studied conditions [40]. Based on the Langmuir model, the maximum adsorption capacities (*q_m_*) of GSNBC for carmine and tartrazine at 45 °C were calculated to be 699.43 and 674.21 mg g^−1^, respectively, which are significantly higher than previously reported values (Table S5). The values for the Freundlich parameter 1/n were all less than 1, suggesting that multilayer physisorption also contributed to the adsorption process [41]. The Temkin model, which accounts for chemisorption and describes a linear decrease in the adsorption heat with surface coverage, yielded relatively low correlation coefficients (*R*^2^ = 0.627–0.939), implying that chemisorption is not the sole governing mechanism [41]. The Dubinin–Radushkevich model was employed to distinguish the adsorption type, and the calculated mean adsorption energy (*E*) values ranged from 0.066 to 5.424 kJ mol^−1^, all of which are below 8 kJ mol^−1^, suggesting the physisorption occurred in the adsorption.

#### 3.2.4. Adsorption Thermodynamics

The thermodynamics of carmine and tartrazine adsorption onto GSNBC were evaluated using the van’t Hoff equation (see Supporting Information S2.4), and the corresponding parameters are listed in Table S4. The Gibbs free energy changes (ΔG^0^) for both carmine and tartrazine were negative at 298, 308, and 318 K, and their negative values increased with increasing temperature, indicating that the adsorption is a spontaneous process and elevated temperature is favorable for adsorption [34]. The ΔG^0^ values ranged from −20 to 0 kJ mol^−1^, and ΔH^0^ was below 20 kJ mol^−1^. The low D-R adsorption energies (E < 8 kJ mol^−1^) and ΔH° values indicated that physisorption played an indispensable role [42]. This synergy between the two modes explains the observed ultrafast kinetics and ultrahigh capacities of GSNBC. The positive values of ΔS^0^ imply an increase in randomness at the solid–liquid interface during adsorption, as structural changes occur on the surfaces of both GSNBC and dye molecules, and the increased disorder and randomness enhance the adsorption performance [43].

#### 3.2.5. Cyclic Regeneration and Potential Application

Recyclability of an adsorbent is crucial for evaluating its suitability for practical applications. As shown in Figure 5i, after five consecutive adsorption–desorption cycles, the adsorption capacity of GSNBC for carmine is 638.12 to 489.07 mg g^−1^, and for tartrazine from 607.00 to 445.00 mg g^−1^. This indicated that GSNBC retained approximately 76.6% and 73.3% of its original capacity for carmine and tartrazine, respectively, confirming its good recyclability and adsorption stability [44]. Moreover, the ethanol desorbent can be recovered by simple distillation to reduce the regeneration cost. To evaluate practical applicability, the real food wastewater test was carried out. The *q*_e_ was 89.95 mg g^−1^ and 73.54 mg g^−1^ of carmine and tartrazine respectively, revealing that GSNBC retains considerable dye adsorption capacity even in complex wastewater matrices (Figure S1). For the production of GSNBC, the ginger straw/NaHCO_3_ mass ratio was 1:1, indicating less consumption of NaHCO_3_. Also, the activation yield, washing loss rate and final yield of GSNBC were 22.5%, 0.8% and 21.7% respectively. Additionally, ginger straw and NaHCO_3_ were low-cost and readily available, further supporting the sustainability and practical value of the proposed material. For the practical application, the efficient recovery of GSNBC from aqueous media after adsorption needs to be further considered.

### 3.3. Adsorption Mechanisms of GSNBC

**Figure 6:**
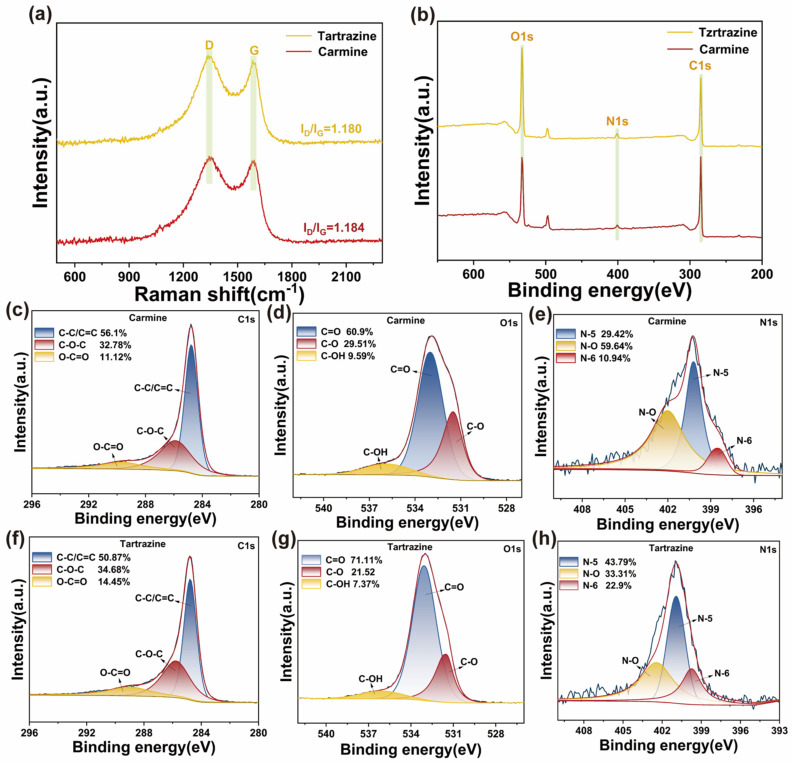
Raman spectra (**a**), X-ray photoelectron spectrometer (XPS) wide scan spectra (**b**); high-resolution XPS spectra of C 1s (**c**,**f**), O 1s (**d**,**g**), N 1s (**e**,**h**) of GSNBC after adsorption.

Structural changes in GSNBC before and after the adsorption of carmine and tartrazine were investigated by Raman spectroscopy and XPS to clarify the adsorption mechanism. After dye adsorption, the Raman spectra (Figure 6a) of both samples still exhibited the characteristic D band (1350 cm^−1^) and G band (1580 cm^−1^) of carbon materials, with I_D_/I_G_ values of 1.184 (carmine) and 1.180 (tartrazine). Both values are close to those before adsorption, indicating that the disordered and defective structure of the carbon skeleton was preserved. The slightly higher I_D_/I_G_ value for carmine suggests stronger π–π interactions with the GSNBC surface, leading to intensified perturbation of the carbon layers [27]. After dye adsorption, the markedly increased proportions of N 1s and O 1s (Figure 6b) indicated the contribution of nitrogen- and oxygen-containing functional groups to dye uptake. For carmine, the relative contents of C–C/C=C, C–O–C, and O–C=O shifted from 66.12%, 18.79%, and 15.09%, respectively, before adsorption to 56.10%, 32.78%, and 11.12%, respectively (Figure 6c); in tartrazine, these contents changed to 50.87%, 34.68%, and 14.45%, respectively (Figure 6f). These variations demonstrate that the abundant functional groups on the GSNBC surface serve as active centers for dye uptake [45]. After carmine adsorption, the C=O content decreased from 82.50% to 60.90%, while the C–O content decreased from 17.50% to 9.57% (Figure 6c). After tartrazine adsorption, the C=O and C–OH contents decreased to 21.52% and 7.37%, respectively (Figure 6g), confirming that C=O and C–OH were the primary functional groups directly involved in adsorption. The newly emerged C–O peak indicates complexation between dye molecules and the material surface, where the functional groups of the dyes coordinate to surface sites, chemically immobilizing the dyes onto the adsorbent surface. After the adsorption of carmine and tartrazine, the relative contents of N-5 and N-6 decreased to 29.42% and 10.94% (carmine) and 43.79% and 22.9% (tartrazine), respectively, whereas the relative content of N–O increased to 59.64% (carmine) and 33.31% (tartrazine). The N-5 and N-6 groups on the GSNBC surface, which contain lone-pair electrons, can act as electron donors that engage in π–π conjugation with the aromatic rings of the dyes, enhancing the adsorption performance. The increase in N–O content may be partially ascribed to the contribution of nitrogen−containing groups such as nitro and sulfonic acid groups from the dyes, and the high electronegativity of these groups enhances electrostatic attraction to dye molecules, thereby promoting adsorption [46].

**Figure 7:**
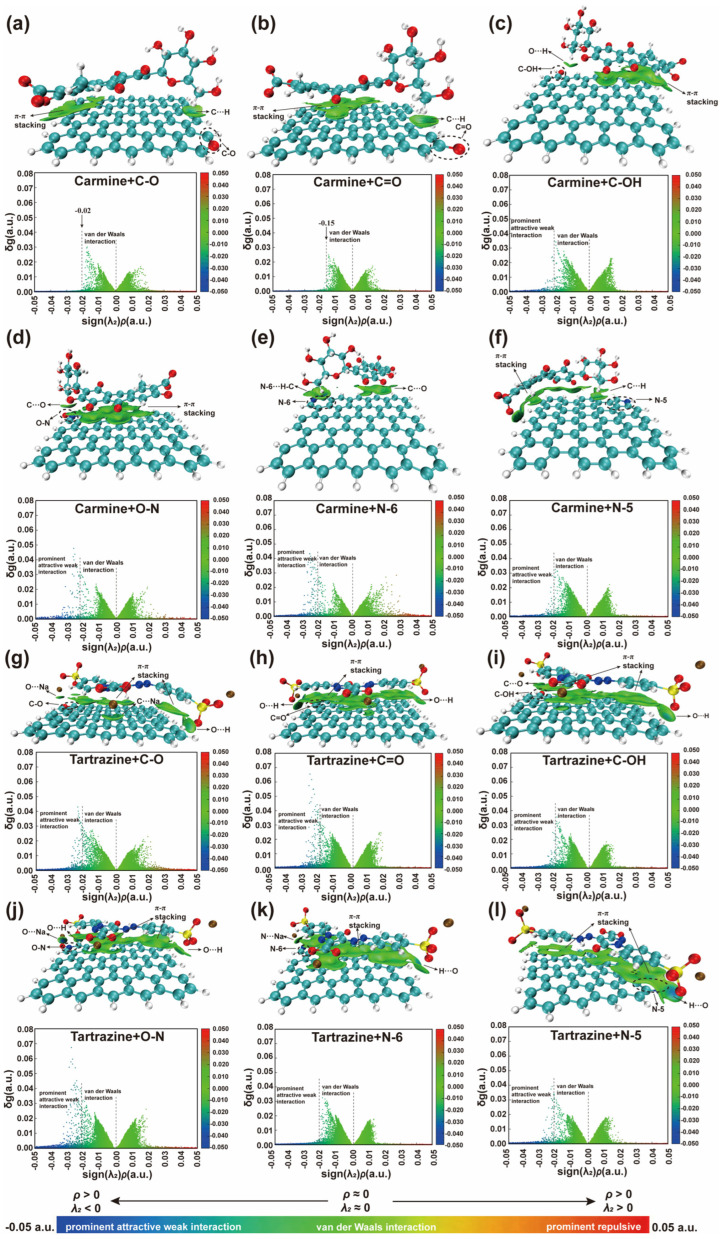
The adsorption mechanism of modified carbon materials to carmine and tartrazine was analyzed by IGMH.C–O group adsorbed carmine (**a**), C=O group adsorbed carmine (**b**), C–OH group adsorbed carmine (**c**), N-O group adsorbed carmine (**d**), N-6 group adsorbed carmine (**e**), N-5 group adsorbed carmine (**f**), C–O group adsorbed tartrazine (**g**), C=O group adsorbed tartrazine (**h**), C–OH group adsorbed tartrazine (**i**), N–O group adsorbed tartrazine (**j**), N-6 group adsorbed tartrazine (**k**), N-5 group adsorbed tartrazine (**l**).

DFT calculations combined with an independent gradient model based on the Hirshfeld partition (IGMH) were employed to visualize the specific inter- and intra-fragment interactions within the chemical system [47], further elucidating the adsorption mechanism of carmine and tartrazine onto GSNBC at the molecular level. Based on the XPS peak-fitting results, functional groups comprising C–O, C=O, C–OH, N–O, N-6, and N-5 were identified. A graphitic carbon cluster consisting of 23 fused aromatic rings was used to represent the structure of the BC. As shown in Figure 7, all the functional group-modified carbon frameworks exhibited significant van der Waals interactions (green isosurfaces) with the dye molecules, accompanied by strong attractive interactions (blue isosurfaces) [47].

For carmine, the interactions of the C–O and C=O groups with the dye were dominated by π–π stacking and C–H contacts (Figure 7a,b). These oxygen-containing functional groups did not achieve effective site-matching adsorption with carmine, owing to positional mismatch or steric hindrance at the adsorption sites [48]. Meanwhile, the van der Waals interaction strength of the C–O structure was greater than that of the C=O structure, corresponding to the peaks around sign(λ_2_)ρ = −0.02 and sign(λ_2_)ρ = −0.15 in the reduced density gradient (RDG) scatter plot, which can be attributed to differences in local electron distribution and polarization response between the two, consequently affecting the intermolecular dispersion forces and the overall van der Waals interactions [17]. As shown in Figure 7c, the C–OH group acted both as a hydrogen-bond donor to form strong O–H···O hydrogen bonds with carmine and simultaneously facilitated π–π stacking between the aromatic fragments of the carbon skeleton and the aromatic system of carmine, giving rise to blue isosurfaces characteristic of strong attractive interactions [49]. In Figure 7d–f, when the edge carbon atoms of the carbon skeleton were substituted by functional groups containing N–O, N-6, and N-5, the electronegative oxygen atom in the N–O group formed C···O attractions with the partially positively charged carbon atoms on the aromatic ring of carmine, accompanied by π–π stacking between the carbon skeleton and the dye [49], corresponding to the strong attractive peaks in the sign(λ_2_)ρ range of −0.02 to −0.05 a.u. in the plot. For N-6, hydrogen bonds were formed with carmine, while the electronegative oxygen atoms in the carmine molecule produced C···O dipole–dipole attractions with the carbon atoms of the skeleton; therefore, this system featured both van der Waals interactions and hydrogen bonds [50]. The N-5 group, lacking accessible lone-pair electrons or positively charged hydrogen, could only form weak C and H contacts and low-intensity π–π stacking with carmine, resulting in either ineffective adsorption or only weak π–π stacking [49].

For tartrazine, Figure 7g reveals that the C–O group exhibited a multi-center synergistic adsorption mode: the negatively charged sulfonic oxygen atoms of tartrazine and specific carbon atoms on the carbon skeleton jointly attracted the sodium ion (Na^+^), forming C···Na^+^ and O···Na^+^ interactions [47]; the highly electronegative oxygen of the C–O group formed C–H···O hydrogen bonds with the hydrogen atoms of the C–H bonds in tartrazine; and π–π stacking occurred simultaneously between the aromatic fragments of the carbon skeleton and the aromatic system of tartrazine [51]. These four types of interactions enabled the system to simultaneously exhibit multiple noncovalent interactions, including electrostatic attraction (ion–dipole), hydrogen bonding, and π–π stacking. The interaction between the C=O group and tartrazine was dominated by strong attractive interactions (Figure 7h), where C=O acted as an excellent hydrogen-bond acceptor to form hydrogen bonds with hydrogen donors in tartrazine, accompanied by face-to-face π–π stacking between the aromatic rings of the carbon skeleton and the conjugated system of tartrazine [51]. The C–OH group served as both a hydrogen-bond donor (O–H) and acceptor (O) (Figure 7i): its highly electronegative oxygen atom accepted the polar hydrogen of tartrazine through lone-pair electrons to form H···O hydrogen bonds, and simultaneously engaged in strong C···O dipole–dipole attractions with the partially positively charged carbon atoms on the aromatic rings [52]. Meanwhile, the large conjugated aromatic system of tartrazine provided an ideal platform for π–π stacking. In Figure 7j, multiple interactions existed between the N and O group and tartrazine, including O···Na^+^ electrostatic interactions. Electronegative O also acted as a hydrogen-bond acceptor, forming hydrogen bonds with polar H atoms. Concurrently, π–π stacking occurred between the aromatic fragments of the attached carbon skeleton and tartrazine. Figure 7k illustrates that the N-6 group achieved adsorption through N···Na^+^ ion–dipole interactions [53], N–H···O hydrogen bonding (where N–H acted as a hydrogen-bond donor to the electronegative oxygen of tartrazine), and π–π stacking between the carbon skeleton and conjugated system of tartrazine. Figure 7l demonstrates that the N-5 group relied on its N–H group to form hydrogen bonds with the electronegative oxygen of tartrazine, synergistically combined with π–π stacking from the aromatic fragments of the carbon skeleton [54].

**Figure 8:**
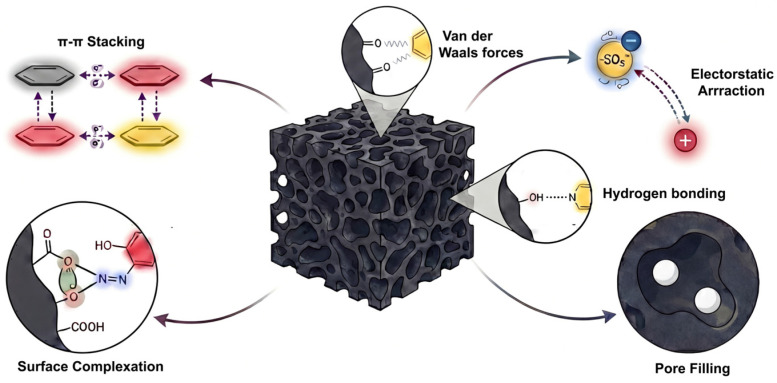
Adsorption mechanism of carmine and tartrazine by GSNBC.

Finally, the adsorption mechanisms of GSNBC for carmine and tartrazine were systematically elucidated (Figure 8). Adsorption kinetics and thermodynamics analyses indicated that the adsorption of two dyes onto GSNBC involved both physisorption and chemisorption. Specifically, the well-developed pore structure and large specific surface area of GSNBC provided abundant active adsorption sites for dye molecules, and pore filling was an important adsorption mechanism. The oxygen- and nitrogen-containing functional groups on the GSNBC surface captured the dye molecules through multiple synergistic interactions including van der Waals forces, electrostatic attraction and surface complexation [55].

## 4. Conclusions

A self-nitrogen-doped porous biochar (GSNBC) was facilely prepared using ginger straw as the precursor and NaHCO_3_ as the mild activator at pyrolysis temperature of 800 °C and ginger straw/NaHCO_3_ mass ratio of 1:1. The NaHCO_3_ activation significantly enhanced the pore structure, specific surface area, and adsorption performance of the biochar toward food dyes including carmine and tartrazine. The as-prepared biochar exhibited rapid and efficient removal of the two dyes and good regeneration stability. The adsorption process followed the pseudo-second-order kinetic and Langmuir isotherm models, indicating that adsorption was predominantly governed by chemical interactions and characterized by monolayer adsorption. The DFT analysis shows that π–π stacking and hydrogen bonding were the main adsorption interactions. Therefore, GSNBC can be employed as a sustainable and efficient adsorbent for treating dye wastewater, offering a new route for the resource utilization of ginger straw.

## Data Availability

The original contributions presented in this study are included in the article. Further inquiries can be directed to the corresponding authors.

## Supplementary Materials

The following supporting information can be downloaded at: https://www.mdpi.com/article/10.3390/foods15152668/s1, Figure S1: Comparison of dye adsorption performance of GSNBC in purified water and real water samples (c0: 700 mg L^−1^, T: 25 °C, t: 60 min). Table S1: Pore textural properties of different proportions of GSNBC samples. Table S2: Parameters of kinetic models for the adsorption of dye by GSNBC. Table S3: The parameters of Langmuir, Freundlich, Temkin, and Dubinin–-Radushkevich models of carmine and tartrazine adsorption by GSNBC. Table S4: Thermodynamic parameters of carmine and tartrazine adsorption by GSNBC. Equations of the formation process of GSNBC. Adsorption model: Kinetic models; isotherm models; adsorption thermodynamic. Table S5: Comparison of dye adsorption properties between this work and previously reported adsorbents [36,56,57,58]. S2.1 Equations of the formation process of GSNBC. S2.2 Equations of adsorption kinetic models S2.3 Equations of adsorption isotherm models. S2.4 Equations of adsorption thermodynamic.
