# Supplementary material for: A Facile Ginger Straw-Based Self-Nitrogen-Doped Biochar Activated by NaHCO3: Fast and Efficient Adsorption Toward Food Dyes of Tartrazine and Carmine
Source: Foods. 2026 Jul 29;15(15):2668. doi: 10.3390/foods15152668 (PMC13464663; doi:10.3390/foods15152668)
Supplement: Supplementary file 1 [file foods-15-02668-s001.zip › foods-4419234-supplementary.pdf]

Supporting information

A Facile Ginger Straw-Based Self Nitrogen-Doped Biochar Activated by $NaHCO_3$: Fast and Efficient Adsorption toward Food Dyes of Tartrazine and Carmine

Mingwan Liu [1,†], Zhuhua Gong [1,†] Zhenghang Guo [1], Yu Yu [1], Shuangshuang Bai [1], Qi Zhang [1], Qinghong Liao [1], Hongjia Lu [1], Honglei Li [1], Yuming You [1,*] and Wenlin Zhang [1,*]

1 Chongqing Key Laboratory for Germplasm Innovation of Special Aromatic Spice Plants, College of Smart Agriculture/Institute of Special Plants, Chongqing University of Arts and Sciences, Chongqing 402160, China

† Contributed equally

* Correspondence: xuxiaojiaoyou@126.com(Y.Y); zhangwenlin@cqwu.edu.cn (W.Z.)

1. Figures and tables

Figures and table captions

Figure S1 Comparison of dye adsorption performance of GSNBC in purified water and real water samples (c_0: 700 mg L^{-1}, T: 25℃, t: 60 min).

Table S1 Pore textural properties of different proportions of GSNBC samples.

Table S2 Parameters of kinetic models for the adsorption of dye by GSNBC.

Table S3 The parameters of Langmuir, Freundlich, Temkin, and Dubinin-Radushkevich models of carmine and tartrazine adsorption by GSNBC.

Table S4 Thermodynamic parameters of carmine and tartrazine adsorption by GSNBC.

Table S5 Comparison of dye adsorption properties between this work and previously reported adsorbents

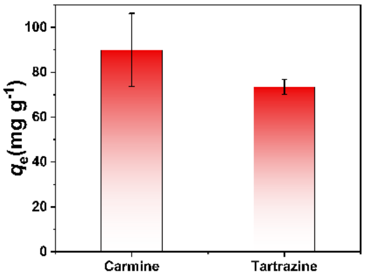

Figure S1. Comparison of dye adsorption performance of GSNBC in purified water and real water samples (c_0: 700 mg L^{-1}, T: 25℃, t: 60 min).

Table S1. Pore textural properties of BC and GSNBC.

Sample	BET area (m^2 g^{-1})	Micropore area (m^2 g^{-1})	Total pore volume (cm^3 g^{-1})	Micropore volume (cm^3 g^{-1})	Average pore size (nm)
BC	57.980	7.113	0.121	0.127	8.380
GSBC-1	1712.517	211.815	1.248	0.091	2.917

Table S2. Parameters of kinetic models for the adsorption of dye by GSNBC.

Adsorption kinetic model	Parameters	Carmine	Tartrazine
Pseudo-first order model	$q_{e\ exp}$ (mg g^{-1})	638.961	601.302
	$q_{e\ cal}$ (mg g^{-1})	637.923	599.213
	k_1 (min^{-1})	5.381	8.492
	R^2	0.881	0.852
Pseudo-second order model	$q_{e\ exp}$ (mg g^{-1})	638.96	601.301
	$q_{e\ cal}$ (mg g^{-1})	641.84	600.072
	k_2 (min^{-1})	0.444	0.225
	R^2	0.973	0.978

Table S3. The parameters of Langmuir, Freundlich, Temkin, and Dubinin-Radushkevich models of carmine and tartrazine adsorption by GSNBC.

Adsorption kinetic model	Parameters	AR			TT		
		25°C	35°C	45°C	25°C	35°C	45°C
Langmuir	q_m (mg g^{-1})	612.52	659.752	699.43	616.47	634.60	674.212
	K_L (L mg^{-1})	3.451	21.752	6.891	0.103	0.372	1464.886
	R^2	0.999	0.922	0.941	0.999	0.999	0.960
Freundlich	K (mg g^{-1} $mg/L)^{-1/n}$	295.085	447.096	431.147	186.675	271.783	522.391
	$1/n$	0.152	0.093	0.104	0.215	0.166	0.061
	R^2	0.783	0.743	0.657	0.854	0.801	0.651
Tenkin	K_T (L mg^{-1})	0.009	1.7865	0.234	0.001	0.007	0.523
	b_T	22.892	36.663	27.961	21.709	23.716	35.897
	R^2	0.939	0.811	0.834	0.811	0.939	0.627
Dubinin-Radushkevich	q_m (mg g^{-1})	604.135	655.756	689.231	604.133	655.743	689.239
	β (mol^2 J^{-2})	2.864	0.066	0.157	2.864	5.583	3.565
	E (kJ mol^{-1})	0.831	5.422	0.351	0.835	0.066	0.157
	R^2	0.894	0.923	0.937	0.894	0.923	0.937

Table S4. Thermodynamic parameters of carmine and tartrazine adsorption by GSNBC.

Dyes	Temperature (K)	$\ln K_D$	ΔG^0 (kJ mol^{-1})	ΔH^0 (kJ mol^{-1})	ΔS^0 (J $(mol \cdot K)^{-1}$)
Carmine	298	0.608	-1.508	0.130	0.507
	308	0.658	-1.686		
	318	0.838	-2.217		
Tartrazine	298	0.411	-1.018	0.193	0.697
	308	0.561	-1.437		
	318	0.751	-1.985		

Table S5. Comparison of dye adsorption properties between this work and previously reported adsorbents

Adsorbent	Dye	V_P(cm^3 g^{-1})	S_{Bet}(m^2 g^{-1})	q_m(mg g^{-1})	Reference
Date palm petiole-based activated carbon	Carmine	0.15	655	53.76	[56]
Sweet potato residue-based biochar	Carmine,tartrazine	1.01	1207	138.06; 264.12	[36]
Activated sawdust-based biochar	Tartrazine	0.62	931	127.72	[57]
Apricot stones-based biochar	Tartrazine	1.26	774	76.80	[58]
Ginger straw-based biochar	Carmine,tartrazine	1.25	1712.5	600.13 ,580.24	This work

2. Equations

S2.1 Equations of the formation process of GSNBC

$$NaHCO_3 \rightarrow Na_2CO_3 + CO_2 + H_2O \quad (S1)$$

$$Na_2CO_3 \rightarrow Na_2O + CO_2 \quad (S2)$$

$$Na_2CO_3 + C \rightarrow Na_2O + CO \quad (S3)$$

$$Na_2O + C \rightarrow Na + CO \quad (S4)$$

S2.2 Equations of adsorption kinetic models

The equations of pseudo-first-order (Eq S5) and pseudo-second-order (Eq S6) models were as follows.

$$q_t = q_e(1-e^{-k_1 t}) \quad (S5) \qquad q_t = \frac{k_2 q_e^2 t}{1+k_2 q_e t} \quad (S6)$$

Among them, q_e (mg g^{-1}) is the adsorption capacity at the equilibrium time, q^t (mg g^{-1}) is the adsorption capacity at a given time, t (min) is the adsorption time, k_1(min^{-1}) and k_2(min^{-1}) are the kinetic rate constants of PFO and PSO, respectively.

S2.3 Equations of adsorption isotherm models

The equations of Langmuir (Eq S7), Freundlich (Eq S8), Temkin (Eq S9) and Dubinin- Radushkevich (Eq S10) models were as follows.

$$q_e = \frac{q_m K_L c_e}{1+K_L c_e} \quad (S7) \qquad q_e = K_F c_e^{1/n} \quad (S8)$$

$$q_e = \frac{RT}{b_T} ln(A_T c_e) \quad (S9) \qquad q_e = q_m exp(-\beta\varepsilon^2) \quad (S10)$$

Among these, q_e (mg g^{-1}) represents the adsorption quantity at the equilibrium time of carmine and tartrazine. q_m(mg g^{-1}) denotes the maximum adsorption capacity of carmine and tartrazine. on GSNBC as determined by the Langmuir model. K_L signifies the Langmuir adsorption constant. c_e (mg g^{-1}) is the equilibrium concentration of carmine and tartrazine solution. K_F signifies the Freundlich adsorption constant. 1/n reflects the adsorption strength. R (8.314 J mol^{-1} K^{-1}) denotes the gas constant, while b_T represents the binding energy. A_T signifies the Temkin adsorption constant. whereas ε denotes the Boltzmann potential, calculable by the equation $\varepsilon = RT$ ln (1+ (1/c_e).

S2.4 Equations of adsorption thermodynamic

The thermodynamic parameters were calculated by (Eq S11-S12).

$$\Delta G = -RT \ln k_e \quad (S11) \qquad \Delta G = \Delta H - T\Delta S \quad (S12)$$

In this context, ΔG represents the change in Gibbs free energy (kJ mol^{-1}), while ke denotes the Freundlich constant at varying temperatures. ΔH represents the enthalpy change (kJ mol^{-1}), whereas ΔS denotes the entropy change (J mol^{-1} K^{-1}), derived from the intercept (ΔH) and slope (ΔS) of the ΔG against T graph.
